# Spontaneous Regression of a Carcinoid Tumor following Pregnancy

**DOI:** 10.1155/2014/481823

**Published:** 2014-12-21

**Authors:** A. Sewpaul, D. Bargiela, A. James, S. J. Johnson, J. J. French

**Affiliations:** ^1^Department of HPB and Transplant Surgery, Newcastle upon Tyne Hospitals NHS Foundation Trust, Freeman Hospital, Newcastle upon Tyne NE7 7DN, UK; ^2^Department of Endocrinology, Newcastle upon Tyne Hospitals NHS Foundation Trust, Royal Victoria Infirmary, Newcastle upon Tyne NE1 4LP, UK; ^3^Department of Cellular Pathology, Newcastle upon Tyne Hospitals NHS Foundation Trust, Royal Victoria Infirmary, Newcastle upon Tyne NE1 4LP, UK

## Abstract

We present a case of spontaneous regression of a neuroendocrine tumor following pregnancy in the absence of chemotherapy, radiotherapy, or alternative medicine (including herbal medicine). The diagnosis of a nonsecretory carcinoid tumor was confirmed using CT imaging, octreotide scan, and histology. Furthermore, serial imaging has demonstrated spontaneous regression of the carcinoid suggesting that pregnancy did not worsen the course of the disease but instead may have contributed to tumour regression. We discuss mechanisms underlying tumour regression and the possible effect of pregnancy on these processes.

## 1. Introduction

Carcinoid tumors were first described more than a century ago [1], but the treatment of patients with advanced disease remains a challenge to clinicians. They are rare endocrine tumors that can develop in several organs in the body. Clinically, patients can have a wide spectrum of signs and symptoms that range from incidental findings of a polyp during endoscopy to the carcinoid syndrome characterized by severe flushing, diarrhea, abdominal cramping, and life-threatening right-sided heart failure [2]. Most carcinoid tumors are indolent but can metastasize to regional lymph nodes and to other organs, including the liver, bone, and the central nervous system [3]. Treatment is determined by tumor location and by the presence of distant metastasis. Surgical resection of the tumor is advocated in patients with localized disease and can often be curative [4]. In certain circumstances surgical resection for metastatic carcinoid tumors (MCTs) can prolong survival [5]. Long-acting somatostatin analogs are effective in providing symptom relief in patients with the carcinoid syndrome [6]. In rare cases a liver transplantation may be proposed, such as young patients with no extrahepatic metastasis and low Ki67 index tumor. The 5-year survival rate is about 49%, with a 5-year survival rate without recurrence of around 25% [7]. Overall, 5- and 10-year survival rates in patients with metastatic disease are favorable, although tumors can be resistant to most forms of medical or surgical therapy [8].

## 2. Case Report

**Figure 1:**
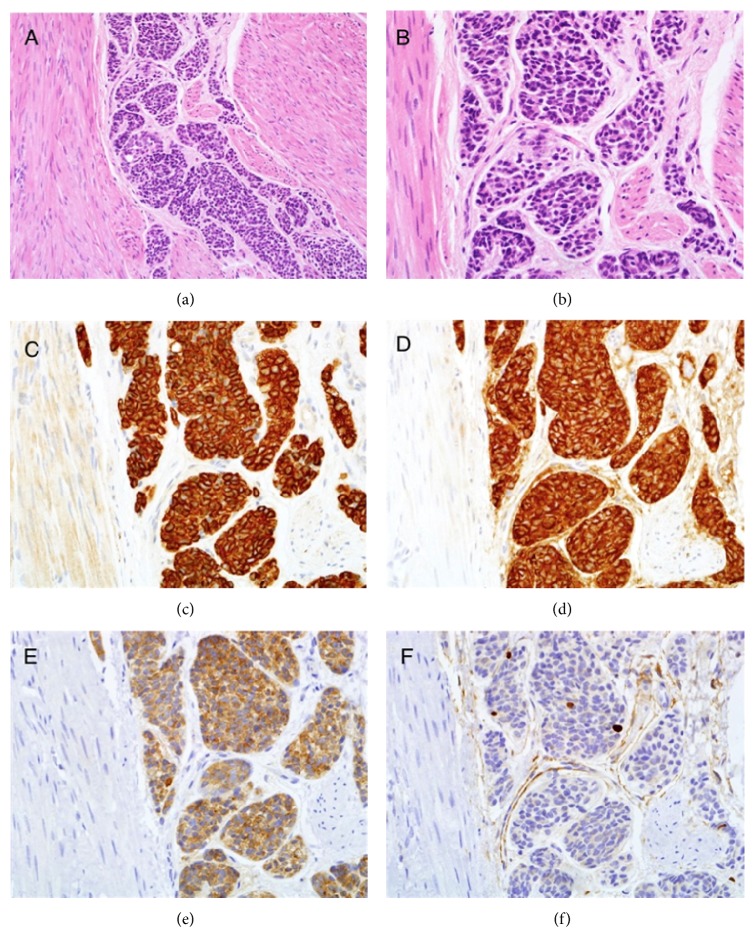
Histology from pelvic mass biopsy sample with a staining pattern in keeping with a carcinoid tumour. (a) Haematoxylin and eosin stain; (b) haematoxylin and eosin stain; (c) epithelial marker, MNF115 positive staining; (d) chromogranin positive staining; (e) synaptophysin positive; (f) Ki67—low index. Magnification—(a) (10x), (b)–(f) (40x).

**Figure 2:**
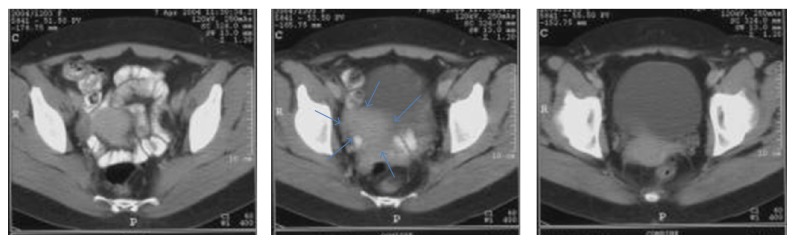
Computed tomography imaging of the pelvis (axial slices) demonstrating an abnormally enhancing right-sided pelvic mass (arrows).

A 35-year-old woman presented to the infertility clinic with secondary infertility. At laparoscopy a pelvic tumour was noted in the right uterosacral region abutting the side wall with spread into the pouch of Douglas. This mass was hard, irregular, and white in appearance and biopsy confirmed a carcinoid tumor with a low Ki-67 index (measure of cell division activity) (grade 1) (Figure 1). She had no symptoms of flushing, diarrhea, or local discomfort. Endocrine screening showed a nonsecretory tumor with normal fasting gut hormones and normal urinary 5H1AA levels. Tumor markers (CEA, CA-125, AFP, and calcitonin) levels were within normal limits. The patient was transferred to a tertiary centre for further investigation and management. A CT scan of her abdomen and pelvis confirmed the right-sided pelvic mass and peritoneal deposits, but the origin of the tumor was difficult to identify, though the appendix was felt to be the presumed origin of disease (Figure 2).

During this period the patient became pregnant. As imaging at that time indicated radiologically static disease, a neuroendocrine tumour multidisciplinary team decision was made to postpone any surgical intervention until after delivery. The patient was monitored by the endocrinology, surgical, and obstetric teams throughout an uneventful antenatal period. A healthy baby was born by elective Caesarean section, with this delivery method chosen to avoid complications from the tumor.

**Figure 3:**
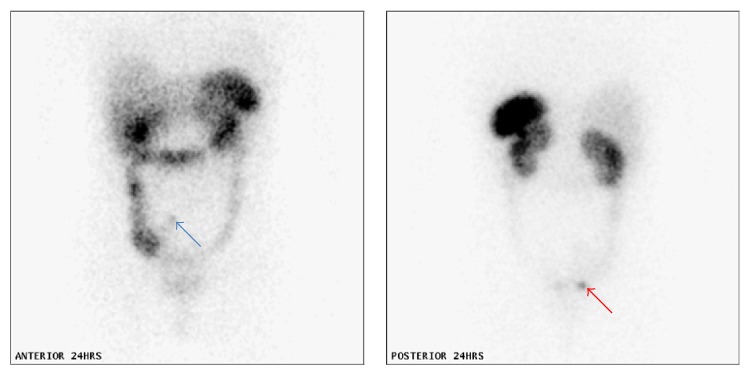
Octreotide scan showing increased tracer uptake persisting on 24-hour images in the right lower pelvis corresponding to the presence of a tumour metastasis (blue arrow) in the pouch of Douglas behind and superior to the urinary bladder. Evidence of persisting focally persistent increased uptake on 24-hour scans in the appendix (red arrow), the presumed primary disease site.

**Figure 4:**
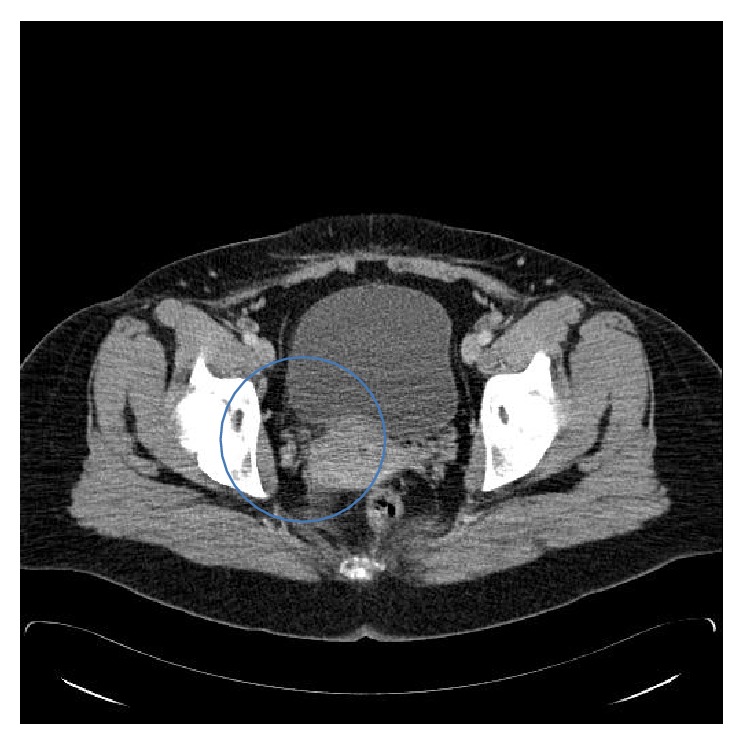
CT at 6 months postpartum. The previously visible right-sided pelvic mass is no longer present.

**Figure 5:**
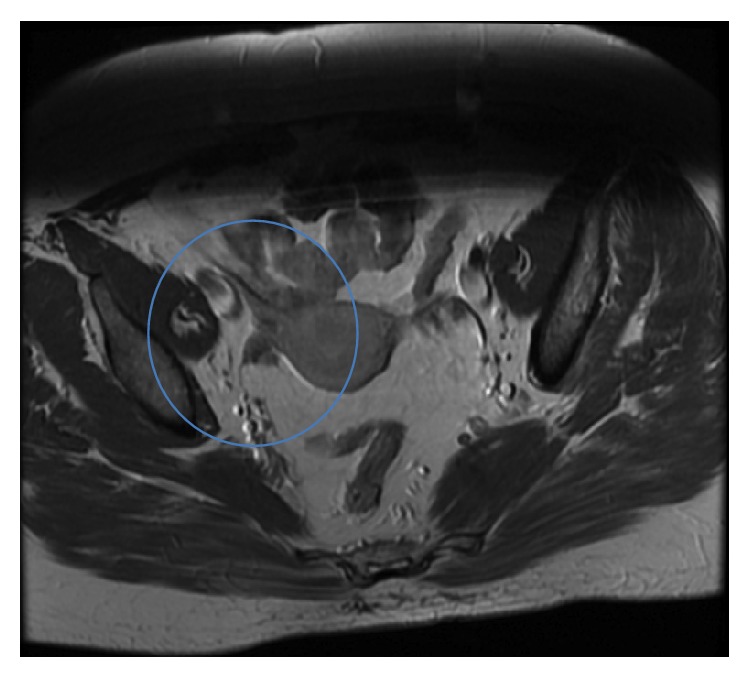
Follow-up MRI of pelvis at 4 years postpartum confirms sustained regression with no signs of tumour recurrence.

Investigations resumed postpartum for the purpose of staging and determining treatment options. An indium^111^ octreotide study, three months postpartum, showed increased tracer uptake in the lower pelvis, correlating to the original tumor as well as a focal increase in the region of the terminal ileum suggesting the location of the primary lesion (Figure 3). Having identified a likely primary site, surgical resection was planned. A repeat CT scan, five months postpartum, was undertaken (Figure 4) which failed to detect the original mass. Following MDT review the decision was for repeat laparoscopy. This demonstrated no macroscopic pelvic or abdominal abnormality. A colonoscopy to the terminal ileum with random biopsies was also normal. Nine years later the patient has remained asymptomatic with no evidence of disease recurrence. Surveillance consisted of two-yearly pelvic MRI imaging. The most recent scan has shown no evidence of abnormality noted in the adnexae bilaterally or around the ileocaecal region suggesting full regression of the previously noted carcinoid tumor (Figure 5). No chemotherapy, radiotherapy, or alternative medicine (including herbal medicine) was given before, during, or after the pregnancy.

## 3. Discussion

The incidence of cancer in pregnancy is rare, complicating between 0.02% and 0.1% of pregnancies [9]. The most common cancers associated with pregnancy are cervical, breast, and ovarian [9, 10], with metastatic carcinoid reported exceedingly rarely [4]. There are no guidelines for the management of carcinoid in pregnancy.

There is only one other reported incidence of spontaneous regression of a neuroendocrine tumour (it was a bronchial carcinoid tumor) following pregnancy [11]. Spontaneous regression of malignant tumours (referred to as spontaneous regression of cancer, SRC) refers to partial or complete disappearance of cancer without medical intervention. It is a very rare phenomenon but has been reported in a variety of cancers, including malignant lymphoma [12, 13], renal cell carcinoma [14, 15], hepatocellular carcinoma [16], and neuroblastoma [17]. Although SRC has often been questioned, the literature describes a number of cases demonstrating this phenomenon. In 1966, Everson and Cole [18] published a classical monograph review of 176 SRC cases published from 1900 to 1964. In 1990, Challis and Stam [19] reported cases from 1900 to 1987, the majority of which occurred in renal cell carcinoma, choriocarcinoma, neuroblastoma, melanoma, breast cancer, and leukemia and lymphomas. Sawant et al. [20] reported on a 40-year-old premenopausal woman who presented with an epigastric lump that was confirmed by open biopsy to be a large gastric carcinoid. The patient had raised serum glucagon level and increased excretion of 5-hydroxyindoleacetic acid in the urine. As she declined a surgical intervention, follow-up at 3 monthly intervals was the agreed action. At 6 months later the tumour had decreased considerably in size and at one year it was no longer palpable and ultrasound examination confirmed that it had completely regressed. Normal levels of urinary 5-HIAA were also noted.

The mechanism driving this exceptional phenomenon remains elusive. The prevalent hypotheses regarding mechanisms leading to spontaneous regression include that of an immunological response in the host [21], activation of proapoptotic pathways [17], cytokines and growth factors [22, 23], and psychological mechanisms [24], all suggested.

Renal cell carcinoma accounts for the largest number of patients with spontaneous regression with both histological and radiological confirmation and thus offers the best system to characterise the immunological response in spontaneous regression [14, 15, 25]. The increased incidence of certain tumors in immunosuppressed individuals and regression following reduction of immunosuppresive agents [26, 27] lends further support to this hypothesis. Cytokines, interferon, and interleukins 2, 6, and 8 (IL-2, IL-6, and IL-8) exert antitumor effects through activation of the immune system [28]. In a study comparing the cytotoxicity of B and T cells against mammary tumour epithelial cells, parous rats were found to demonstrate significantly increased tumouricidal activity compared to virgin controls. The suggested mechanism was mediated cytotoxic T lymphocytes (CTL) primed by pregnancy-specific antigens in the parous host [29]. Furthermore, natural killer (NK) cells have also been shown to have a spontaneous cytotoxic effect on virus infected and malignant cells [30] and NK activity is known to increase during pregnancy [31].

Another potential mechanism involves vascular endothelial growth factor (VEGF) receptor blockade leading to rapid, robust, and progressive regression of tumour vasculature, increased intratumoural hypoxia, apoptosis, and reduced tumour invasiveness and metastasis as seen in pancreatic islet cancer [32]. This proapoptotic process is thought to be driven by both oncogene and tumour suppressor gene and has been suggested as a putative mechanism of SRC in leukemia [33].

In this report, we describe the second reported case of spontaneous regression of a neuroendocrine tumor following pregnancy in the absence of chemotherapy, radiotherapy, or alternative medicine (including herbal medicine). The diagnosis of a nonsecretory carcinoid tumor was confirmed using CT imaging, octreotide scan, and histology. Furthermore, serial imaging has demonstrated spontaneous regression of the carcinoid suggesting that pregnancy did not worsen the course of the disease but instead may have contributed to tumor regression. The mechanisms underlying tumor regression and the effect of pregnancy on these processes are interesting and further study may be helpful in guiding future therapeutic targets for the treatment of cancer.

## References

[1] Lubarsch O. (1888). Ueber den primären Krebs des Ileum nebst Bemerkungen über das gleichzeitige Vorkommen von Krebs und Tuberculose. *Archiv für Pathologische Anatomie und Physiologie und für Klinische Medicin*.

[2] Kulke M. H., Benson A. B., Bergsland E. (2012). Neuroendocrine tumors. *Journal of the National Comprehensive Cancer Network*.

[3] Kulke M. H., Mayer R. J. (1999). Carcinoid tumors. *The New England Journal of Medicine*.

[4] Modlin I. M., Lye K. D., Kidd M. (2003). A 5-decade analysis of 13,715 carcinoid tumors. *Cancer*.

[5] Coan K. E., Gray R. J., Schlinkert R. T., Pockaj B. A., Wasif N. (2013). Metastatic carcinoid tumors—are we making the cut?. *The American Journal of Surgery*.

[6] Öberg K. E. (2012). The management of neuroendocrine tumours: current and future medical therapy options. *Clinical Oncology*.

[7] Gedaly R., Daily M. F., Davenport D., McHugh P. P., Koch A., Angulo P., Hundley J. C. (2011). Liver transplantation for the treatment of liver metastases from neuroendocrine tumors: an analysis of the UNOS database. *Archives of Surgery*.

[8] Alsina M., Marcos-Gragera R., Capdevila J., Buxó M., Ortiz R. M., Barretina P., Vilardell L., Brunet J., Beltran M., Izquierdo À. (2011). Neuroendocrine tumors: a population-based study of incidence and survival in Girona province, 1994–2004. *Cancer Epidemiology*.

[9] Lishner M. (2003). Cancer in pregnancy. *Annals of Oncology*.

[10] Pereg D., Koren G., Lishner M. (2008). Cancer in pregnancy: gaps, challenges and solutions. *Cancer Treatment Reviews*.

[11] Luosto R., Koikkalainen K., Sipponen P. (1974). Spontaneous regression of a bronchial carcinoid tumour following pregnancy. *Annales Chirurgiae et Gynaecologiae Fenniae*.

[12] Mizuno T., Ishigaki M., Nakajima K., Matsue T., Fukushima M., Minato H., Nojima N., Atsushi S., Ishigami K., Atsumi H., Ito T., Iguchi M., Usuda D., Okamura H., Urashima S., Asano M., Fukuda A., Izumi Y., Takekoshi N., Kanda T. (2013). Spontaneous remission of epstein-barr virus-positive diffuse large b-cell lymphoma of the elderly. *Case Reports in Oncology*.

[13] Mohsen A., Ghanem H., El-Bayoumi J., Tabbara I. (2012). Spontaneous regression of classical hodgkin lymphoma: a case report and review of the literature. *Clinical Advances in Hematology and Oncology*.

[14] Janiszewska A. D., Poletajew S., Wasiutyński A. (2013). Spontaneous regression of renal cell carcinoma. *Contemporary Oncology*.

[15] Abubakr Y. A., Chou T.-H., Redman B. G. (1994). Spontaneous remission of renal cell carcinoma: a case report and immunological correlates. *Journal of Urology*.

[16] Oquinena S., Guillen-Grima F., Iñarrairaegui M., Zozaya J. M., Sangro B. (2009). Spontaneous regression of hepatocellular carcinoma: a systematic review. *European Journal of Gastroenterology & Hepatology*.

[17] Pritchard J., Hickman J. A. (1994). Why does stage 4s neuroblastoma regress spontaneously?. *The Lancet*.

[18] Everson T. C., Cole W. H. (1966). *Spontaneous Regression of Cancer*.

[19] Challis G. B., Stam H. J. (1990). The spontaneous regression of cancer. A review of cases from 1900 to 1987. *Acta Oncologica*.

[20] Sawant P. D., Nanivadekar S. A., Shroff C. P., Srinivas A., Dewoolkar V. V. (1989). Spontaneous regression of large gastric carcinoid. *Indian Journal of Gastroenterology*.

[21] Jessy T. (2011). Immunity over inability: the spontaneous regression of cancer. *Journal of Natural Science, Biology and Medicine*.

[22] Balkwill F. R., Naylor M. S., Malik S. (1990). Tumour necrosis factor as an anticancer agent. *European Journal of Cancer*.

[23] Broder S., Karp J. E. (1995). Progress against cancer. *Journal of Cancer Research and Clinical Oncology*.

[24] Papac R. J. (1998). Spontaneous regression of cancer: possible mechanisms. *In Vivo*.

[25] Kavoussi L. R., Levine S. R., Kadmon D., Fair W. R. (1986). Regression of metastatic renal cell carcinoma: a case report and literature review. *The Journal of Urology*.

[26] Morton L. M., Gibson T. M., Clarke C. A., Lynch C. F., Anderson L. A., Pfeiffer R., Landgren O., Weisenburger D. D., Engels E. A. (2014). Risk of myeloid neoplasms after solid organ transplantation. *Leukemia*.

[27] Gorshtein A., Gross D. J., Barak D., Strenov Y., Refaeli Y., Shimon I., Grozinsky-Glasberg S. (2012). Diffuse idiopathic pulmonary neuroendocrine cell hyperplasia and the associated lung neuroendocrine tumors: clinical experience with a rare entity. *Cancer*.

[28] Schwaab T., Schwarzer A., Wolf B., Crocenzi T. S., Seigne J. D., Crosby N. A., Cole B. F., Fisher J. L., Uhlenhake J. C., Mellinger D., Foster C., Szczepiorkowski Z. M., Webber S. M., Schned A. R., Harris R. D., Barth R. J., Heaney J. A., Noelle R. J., Ernstoff M. S. (2009). Clinical and immunologic effects of intranodal autologous tumor lysate-dendritic cell vaccine with aldesleukin (interleukin 2) and IFN-*α*2a therapy in metastatic renal cell carcinoma patients. *Clinical Cancer Research*.

[29] Chakravarty P. K., Sinha D. K. (1992). Pregnancy-induced antitumor cytotoxicity of T cell-rich fraction against mammary adenocarcinoma cells in rats. *Carcinogenesis*.

[30] Herberman R. B., Ortaldo J. R. (1981). Natural killer cells: their roles in defenses against disease. *Science*.

[31] Manaseki S., Searle R. F. (1989). Natural killer (NK) cell activity of first trimester human decidua. *Cellular Immunology*.

[32] You W.-K., Sennino B., Williamson C. W., Falcón B., Hashizume H., Yao L.-C., Aftab D. T., McDonald D. M. (2011). VEGF and c-Met blockade amplify angiogenesis inhibition in Pancreatic Islet Cancer. *Cancer Research*.

[33] Matsushita K., Arima N., Fujiwara H. (1999). Spontaneous regression associated with apoptosis in a patient with acute-type adult T-cell leukemia. *The American Journal of Hematology*.

